# Synthesis of Novel Fluorinated Xanthine Derivatives with High Adenosine A_2B_ Receptor Binding Affinity

**DOI:** 10.3390/ph14050485

**Published:** 2021-05-19

**Authors:** Marcel Lindemann, Sladjana Dukic-Stefanovic, Sonja Hinz, Winnie Deuther-Conrad, Rodrigo Teodoro, Cathleen Juhl, Jörg Steinbach, Peter Brust, Christa E. Müller, Barbara Wenzel

**Affiliations:** 1 Helmholtz-Zentrum Dresden-Rossendorf, Institute of Radiopharmaceutical Cancer Research, Department of Neuroradiopharmaceuticals, 04318 Leipzig, Germany; s.dukic-stefanovic@hzdr.de (S.D.-S.); w.deuther-conrad@hzdr.de (W.D.-C.); r.teodoro@hzdr.de (R.T.); Steinbach-joerg@web.de (J.S.); p.brust@hzdr.de (P.B.); b.wenzel@hzdr.de (B.W.); 2 ROTOP Pharmaka GmbH, 01328 Dresden, Germany; service@rotop-pharmaka.de; 3 Pharma Center Bonn, Pharmaceutical Institute, Pharmaceutical & Medicinal Chemistry, University of Bonn, 53121 Bonn, Germany; Sonja.Hinz@uni-wh.de (S.H.); christa.mueller@uni-bonn.de (C.E.M.)

**Keywords:** xanthine, adenosine A_2B_ receptor, adenosine, PSB-603

## Abstract

The G protein-coupled adenosine A_2B_ receptor is suggested to be involved in various pathological processes accompanied by increased levels of adenosine as found in inflammation, hypoxia, and cancer. Therefore, the adenosine A_2B_ receptor is currently in focus as a novel target for cancer therapy as well as for noninvasive molecular imaging via positron emission tomography (PET). Aiming at the development of a radiotracer labeled with the PET radionuclide fluorine-18 for imaging the adenosine A_2B_ receptor in brain tumors, one of the most potent and selective antagonists, the xanthine derivative PSB-603, was selected as a lead compound. As initial biodistribution studies in mice revealed a negligible brain uptake of [^3^H]PSB-603 (SUV_3min_: 0.2), structural modifications were performed to optimize the physicochemical properties regarding blood–brain barrier penetration. Two novel fluorinated derivatives bearing a 2-fluoropyridine (**5**) moiety and a 4-fluoro-piperidine (**6**) moiety were synthesized, and their affinity towards the four adenosine receptor subtypes was determined in competition binding assays. Both compounds showed high affinity towards the adenosine A_2B_ receptor (*K*_i_ (**5**) = 9.97 ± 0.86 nM; *K*_i_ (**6**) = 12.3 ± 3.6 nM) with moderate selectivity versus the other adenosine receptor subtypes.

## 1. Introduction

Adenosine is an important signaling molecule that is released from cells or generated extracellularly after the cleavage of adenosine 5’-triphosphate (ATP) by ectonucleotidases. Extracellular adenosine activates G protein-coupled receptors, which are classified into four subtypes: adenosine A_1_, A_2A_, A_2B_, and A_3_ receptors [1,2,3,4]. Compared to the other subtypes, the adenosine A_2B_ receptor (A_2B_ receptor) has a low expression under physiological conditions and is only activated at higher adenosine concentrations [1,5]. Such high levels can be reached under pathophysiological conditions (e.g., inflammation, cancer, infection, and hypoxia) [6].

Due to its involvement in tumor growth, angiogenesis, metastasis, and immunomodulation, the A_2B_ receptor is an interesting target for the imaging and treatment of cancer [7,8]. It shows an increased expression in various solid tumors and tumor cell lines relative to normal tissues. These findings can be an outcome of the hypoxic conditions and is associated with increased adenosine concentrations in the tumor microenvironment [9,10,11]. 

To date, numerous different antagonists targeting this adenosine receptor subtype have been developed [8,12]. One of the most specific and potent antagonists is the xanthine derivative PSB-603 [13] (see Table 1), which was recently shown to suppress the γ-irradiation-induced translocation of the epidermal growth factor receptor (EGFR) triggering DNA damage repair and radioresistance of tumors. These studies showed a significant involvement of the adenosine A_2B_ receptor by suppression of EGFR translocation and reduction of tumor growth in mice due to pretreatment with PSB-603 [14,15].

One important noninvasive in vivo imaging tool to diagnose cancer and to follow up on therapy responses is positron emission tomography (PET). For this technique, a specific radiotracer with high affinity is needed, which is labeled with a positron-emitting radionuclide, such as carbon-11 (t_1/2_ = 20.4 min) or fluorine-18 (t_1/2_ = 109.8 min). As the main objective of our work was to characterize the expression of the A_2B_ receptor using PET, the development of a specific radioligand labeled with fluorine-18 would support the investigation of this promising target in vitro and in vivo. The examination of the contribution of this receptor subtype in brain tumors was of particular interest to us. 

**Figure 1:**
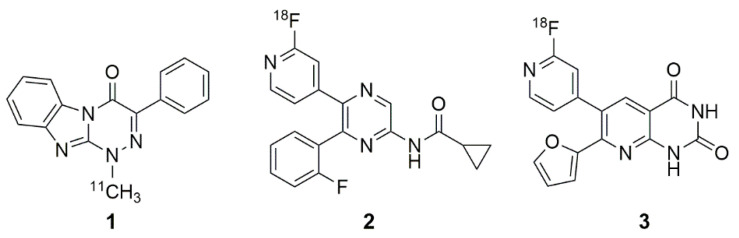
Carbon-11 (**1**) and fluorine-18 (**2** and **3**)-labeled radiotracers for PET imaging of the adenosine A_2B_ receptor.

The first PET radiotracer for imaging of the adenosine A_2B_ receptor was published by Petroni et al. [16] (see Figure 1). The carbon-11-labeled triazinobenzimidazole compound **1** showed only a moderate affinity (IC_50_ (A_2B_) = 210.2 ± 12.3 nM [16]). Its pharmacokinetic properties were investigated in rats. The authors concluded that the radiotracer provides potential for the development of more potent and selective A_2B_ receptor PET imaging tools [16]. The first fluorine-18-labeled radiotracer **2** (see Figure 1) was published in 2018 by our group and showed good affinity towards the adenosine A_2B_ receptor with moderate selectivity towards the other adenosine receptor subtypes [17]. However, metabolism studies in mice revealed a rapid metabolic degradation of **2**. One of the radiometabolites was found to be the cleavage product of the amide bond, resulting in an amino derivative that was able to penetrate the blood–brain barrier (BBB). Recently, we developed the bicyclic radiotracer **3** with a pyridopyrimidine-2,4-dione core (Figure 1) [18]. This compound showed good affinity and selectivity towards the A_2B_ receptor with considerably improved in vivo stability; however, its brain uptake was rather low. 

In recent years, high affinity and selectivity of xanthine-based compounds have been achieved by several groups, resulting in a wide range of structural diversity [8,13,19,20,21,22,23]. One important structural modification of xanthines is *N*-alkylation at the nitrogen atoms *N*1 and *N*3 (see Table 1). The most promising results were obtained with ethyl or propyl substitutions at position *N*1 [23]. Moreover, various substitutions in position 8 of the xanthine core also resulted in compounds with high affinity and selectivity, such as CVT-6975 [20], MRS-1754 [19], PSB-603 [13], and PSB-1901 [23] (see Table 1). All of the substituents contain phenyl or heteroaromatic moieties, of which the phenylsulfonamide-based derivatives show the highest affinity towards the adenosine A_2B_ receptor [23]. The most potent and selective xanthine-based compounds are PSB-603 and PSB-1901, which were published by the Müller group in 2009 [13] and 2019 [23]. They are characterized by propyl substitution on position *N*1 and the phenylsulfonamide, substituted with a 4-chlorophenyl (PSB-603) or 4-bromophenyl (PSB-1901) piperazine moiety, respectively (Table 1).

**Table 1:** Known high-affinity xanthine-based ligands for the A_2B_ receptor (all selectivity ratios *K*_i_(A_x_)/*K*_i_(A_2B_) >210).

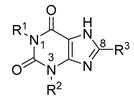	**R^1^**	**R^2^**	**R^3^**	***K*_i_ (*human* A_2B_) ± SEM in nM**
PSB-603 (X = –Cl) PSB-1901 (X = –Br)	–propyl	–H	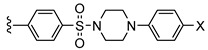	0.553 ± 0.103 [13] ^a^ 0.0835 ± 0.0033 [23] ^a^
MRS-1754	–propyl	–propyl	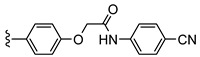	1.97 ± 0.31 [19] ^b^
CVT-6975	–methyl	–methyl	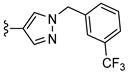	1.0 [20] ^c^

^a^ Determined versus [^3^H]PSB-603. ^b^ Determined versus [^3^H]ZM214385 or [^125^I]IABOPX. ^c^ Determined versus [^3^H]ZM214385.

**Scheme 1:**
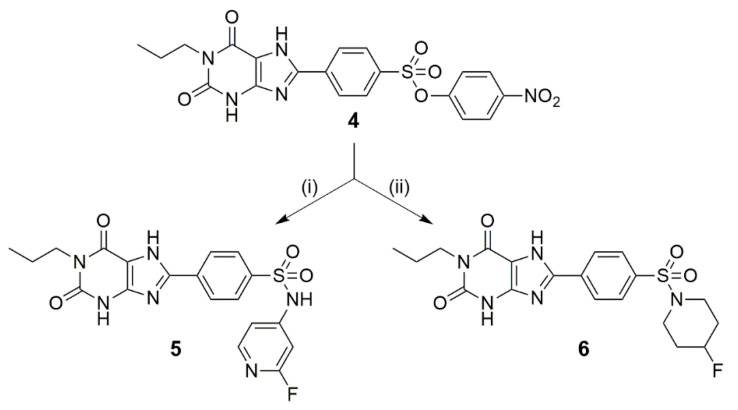
Synthetic procedure for the preparation of the new fluorinated xanthine derivatives **5** and **6**. (i) 2-fluoropyridine-4-amine, NaH, DMF, room temperature, 30 min followed by heating in DMF, 125 °C, 12 h; (ii) 4-fluoropiperidine TFA salt, TEA, DMF, 125 °C, 6.5 h.

Based on these structure–activity relationship results, PSB-603 was used in this study as lead compound for the development of a fluoro-containing PET radiotracer. Initial biodistribution studies in mice were performed to investigate whether [^3^H]PSB-603 would be able to penetrate the BBB. The measured standardized uptake value (SUV) of 0.2 at 3 min p.i. (results are in Section 2.1) revealed that the brain uptake was insufficient for a suitable PET radiotracer for brain imaging [24,25]. Therefore, structural modifications of the PSB-603 lead compound (M = 529 g/mol, clogP = 4.9) were envisaged to improve the brain uptake. Accordingly, the molar mass was reduced by shortening the phenylpiperazinyl moiety, which additionally contributed to a reduction in the high lipophilicity of PSB-603. Furthermore, in order to allow labeling with fluorine-18, fluoropyridine and 4-fluoropiperidine rings were selected as suitable moieties as we have previously shown [17,18,26]. Based on this strategy, the two novel xanthine derivatives **5** and **6** (see Scheme 1) demonstrated, with a molar mass of 444 and 435 g/mol and clogP values of 2.7 and 3.0, respectively, improved physicochemical properties regarding the potential to sufficiently penetrate the BBB for PET imaging. In particular, the clogP values were in the range of 1 to 4, which has been reported to be beneficial for a passive diffusion of molecules through the BBB [27,28,29]. 

## 2. Results and Discussion

### 2.1. Organ Distribution of [^3^H]PSB-603 in Mice

To estimate the ability of [^3^H]PSB-603 to penetrate the BBB in vivo, an initial biodistribution study in healthy CD1 mice was performed. 

For mouse brain, an SUV of 0.21 ± 0.07 (*n* = 3) at 3 min p.i. of [^3^H]PSB-603 was determined, indicating a low uptake of the radioligand. 

The moderate uptake in the pituitary gland corresponds to the higher expression pattern of adenosine A_2B_ receptors, as shown by Rees et al. [30]. However, it may also be explained by the fact that the pituitary gland lacks a BBB. 

**Figure 2:**
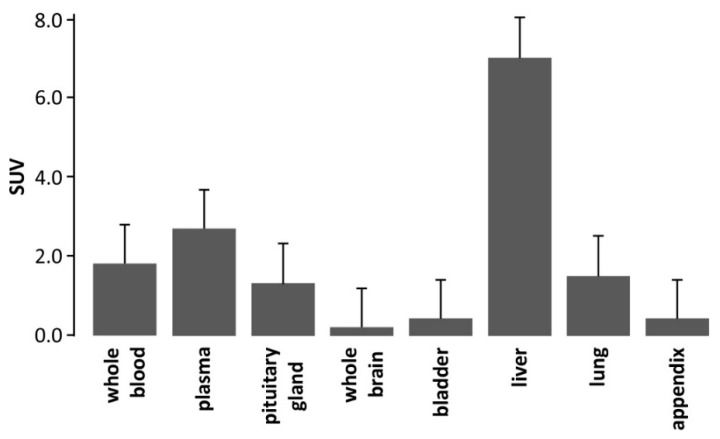
Organ distribution of [^3^H]PSB-603 in mice at 3 min p.i. (data are presented as mean values ± SD; *n* = 3).

Although, RT-PCR studies suggest widespread distribution of the A_2B_ receptor with the highest gene expression in proximal colon, lung, uterus, eye, and bladder [31], the uptake of [^3^H]PSB-603 observed in the bladder, appendix, and lung was low to moderate (Figure 2). The high uptake in the liver is likely related to the metabolic degradation of [^3^H]PSB-603 in this organ. 

### 2.2. Synthesis of ***5*** and ***6***

The synthesis of the new derivatives **5** and **6** was performed as depicted in Scheme 1. The required precursor **4** was synthesized following procedures published in the literature (see Scheme S1 in Supplementary Materials) [32,33,34,35,36].

Yan et al. [37] described the synthesis of **7** (for the structure, see Table 2), where they used aniline as amine in a dimethylformamide (DMF)/pyridine mixture. In adaption to the published conditions, DMF was chosen with an excess amount of 2-fluoropyridin-4-amine under thermal heating (125 °C). Because of the low reactivity of the employed aminopyridine, sodium hydride was used as a strong base. After an acidic workup and chromatographic purification, compound **5** was obtained with a yield of 51% (Scheme 1, NMR and MS spectra in Figures S1–S4 in Supplementary Materials).

The synthesis of **6** was performed according to Yan et al. [37] and Borrmann et al. [13] in DMF with an excess of 4-fluoropiperidine as amine, which was synthesized via a two-step synthetic route [38]. This amine could be directly applied in the form of its trifluoroacetic acid (TFA) salt because the addition of an excess of triethylamine (TEA) to the reaction mixture resulted in the corresponding free base being formed in situ. At a temperature of 125 °C, **6** could be obtained with a yield of 44% after purification (Scheme 1, NMR and MS spectra in Figures S5–S8 in Supplementary Materials).

### 2.3. Affinity towards Adenosine Receptor Subtypes

For the two novel fluorinated xanthine derivatives, the binding affinity towards the adenosine receptor subtypes A_2B_, A_2A_, A_1_, and A_3_ was determined in competition binding assays by incubation of the appropriate standard radioligand with membrane preparations of Chinese hamster ovary (CHO) and human embryonic kidney (HEK) cells, respectively, expressing the corresponding human receptor subtype (Table 2). 

Both compounds showed high affinity towards the adenosine A_2B_ receptor (*K*_i_ (**5**) = 9.97 ± 0.86 nM; *K*_i_ (**6**) = 12.3 ± 3.6 nM), which was slightly increased compared to the nonfluorinated derivatives **7** and **8** (Table 2) reported by Yan et al. [37] and Borrmann et al. [13]. 

The selectivity of **5** and **6** towards the two adenosine receptor subtypes A_1_ and A_3_ were increased compared to those of the nonfluorinated derivatives **7** and **8**. Regarding the adenosine A_2A_ receptor, the introduction of an electron-withdrawing functionality at the piperidine ring, such as the fluorine atom in **6**, showed a negative effect on the selectivity, while the introduction of electron-donating nitrogen in **5** showed an improvement of the selectivity compared to aryl compound **7**.

## 3. Materials and Methods

### 3.1. Organ Distribution Studies of [^3^H]PSB-603 in Mice

All animal studies followed the international guidelines on animal care, and the study protocols were approved by the Landesdirektion Leipzig, the local authority for animal care (Reg.-Nr.: TVV 08/13; Reference number: 24-9168.11/18/8; approval date: 12 June 2013). Animal experiments were performed with female CD-1 mice, 10–12 weeks old, obtained from the Experimental Centre of the Faculty of Medicine (MEZ) at the Leipzig University, Leipzig, Germany.

[^3^H]PSB-603 (specific activity 2.92 TBq/mmol, Pharmaron formerly Quotient Bioresearch (Radiochemicals) Ltd, Cardiff, UK; the precursor was synthesized at the University of Bonn, Germany [13]) was dissolved in 100 µL of sterile isotonic saline and administered as a bolus injection via the tail vein of the restrained animal. At 3 min (*n* = 3; dose: 0.18 ± 0.02 µM), retro-orbital blood samples were obtained from the anesthetized animals. Immediately afterwards, organs of interest were isolated and blood plasma was obtained by centrifugation of the whole blood sample (15,000 rpm, room temperature, 1 min). All samples were weighed and dissolved in SOLVABLE (PerkinElmer Life and Analytical Sciences, Rodgau, Germany) for 5 h at 50 °C, overnight at 30 °C, and a further 5 h at 50 °C. By adding H_2_O_2_, decolorization of the samples was achieved. The samples were left to cool down to room temperature before the addition of 15 mL of Ultima Gold cocktail (PerkinElmer Life and Analytical Sciences, Rodgau, Germany). The samples were shaken overnight, and the radioactivity was measured in a Beckman Liquid Scintillation Counter LS 6500 (Beckman Coulter, Fullerton, CA, USA). For each sample, the percentage of the injected dose was calculated and normalized according to the tissue weight (% ID/g tissue), followed by normalization according to the injected dose and the weight of the animal [(% ID/g tissue)/(100%/g animal)] to calculate the standardized uptake value (SUV) for each tissue.

### 3.2. Chemistry

#### 3.2.1. General Methods and Materials

Analysis of all compounds was performed by MS, HPLC, TLC, and NMR spectroscopy. The spectra (^1^H, ^13^C, ^19^F-NMR, and MS) for the final compounds are given in the Supplementary Materials.

High-resolution mass spectra were recorded on an ESI-qTOF Impact II (Bruker Daltonik GmbH) and an ESI-TOF micrOTOF (Bruker Daltonik GmbH) using ElectroSpray Ionization (ESI). NMR spectra (^1^H, ^13^C, ^13^C-APT, ^19^F, H,H-COSY, HSQC, and HMBC) were recorded on spectrometers from Varian (MERCURY plus 300 MHz and MERCURY plus 400 MHz) and Bruker (AVANCE III HD 400 MHz, DRX-400 400 MHz, and Fourier 300 300 MHz). Splitting patterns were designated as follows: s, singlet; d, doublet; bs, broad singlet; m, multiplet; t, triplet; dd, doublet of doublet; td, triplet of the doublet; and dt, doublet of the triplet. 

Analytical thin-layer chromatography was performed on silica gel-coated plates (Macherey-Nagel, ALUGRAM SIL G/UV254). The spots were identified using a UV lamp (254 nm) or by spraying a solution of 0.1% ninhydrin in ethanol/water 1/10 or dipping into a KMnO_4_ solution (3 g KMnO_4_, 20 g K_2_CO_3_, 0.25 mL glacial acid, 300 mL water). The retention factor (R_f_) is the ratio of the distance of the compound to the starting point and the total distance of the solvent front from the starting point. For purification of the final products, flash column chromatography was used with silica gel ZEOsorb 60/40–63 μm from Apollo Scientific Ltd. and silica gel 40–63 μm from VWR Chemicals. The chemical purity of the final compounds was ≥96% and was controlled by HPLC using a 150 × 3 mm Reprosil-Pur Basic HD 3 μm column (Dr. Maisch GmbH, Germany). These analytical chromatographic separations were performed on a Dionex Ultimate 3000 system incorporating an LPG-3400SD pump, an autosampler WPS-3000 TSL, a column compartment TCC-3000SD, a diode array detector DAD3000 (monitoring from 254 to 720 nm), and a low-resolution mass spectrometer MSQ 3000 (Thermo Fisher Scientific Inc., Waltham, MA, USA). A mixture of acetonitrile (ACN) and aqueous 20 mM NH_4_OAc was used as eluent in a linear gradient system (0–5 min at 25% ACN, 5–45 min up to 95% ACN, 45–55 min at 95% ACN, 55–57 min up to 25% ACN, and 57–65 min at 25%) with a flow of 0.6 mL/min.

All clogP values and chemical names of compounds were generated by ChemDraw Professional 19.0.1.28.

#### 3.2.2. Syntheses

##### 4-(2,6-Dioxo-1-propyl-2,3,6,7-tetrahydro-1*H*-purin-8-yl)-*N*-(2-fluoropyridin-4-yl)benzenesulfonamide (**5**)

In 1 mL DMF 59 mg (0.53 mmol, 5.0 eq.) 4-amino-2-fluoropyridine was dissolved. Then, then 23 mg (0.58 mmol, 5.5 eq.) sodium hydride (60% in paraffin oil) was added, and the reaction mixture was stirred at room temperature for 30 min. After the addition of 50 mg (0.11 mmol, 1.0 eq.) **4** in 1 mL DMF, the reaction mixture was stirred at 125 °C for 12 h, quenched in 100 mL 1 M aq. HCl followed by extraction with 3 × 25 mL ethyl acetate. The combined organic layers were dried over sodium sulfate, filtered and the solvent was removed under reduced pressure. The crude material was purified by column chromatography (DCM/MeOH/HOAc 99:1:1 *v/v/v*, silica gel) to yield 24 mg (0.05 mmol, 51%) of **5**.

R_f_ = 0.07 (DCM/MeOH/HOAc 99:1:1 *v*/*v*/*v*, silica gel). ^1^H NMR (DMSO-d_6_, 300 MHz) δ = 0.85 (t, *J* = 7.4 Hz, 3 H), 1.49-1.61 (m, 2 H), 3.79 (d, *J* = 7.6 Hz, 2 H), 6.36 (d, *J* = 1.8 Hz, 1 H), 6.64 (dt, *J* = 5.8, 1.9 Hz, 1 H), 7.63 (d, *J* = 5.8 Hz, 1 H), 7.84 (d, *J* = 8.5 Hz, 2 H), 8.13 (d, *J* = 8.5 Hz, 2 H) ppm. ^13^C NMR (DMSO-d6, 75 MHz) δ: 11.6, 21.3, 41.9, 97.2 (d, *J* = 39.7 Hz), 108.7, 114.1 (d, *J* = 2.8 Hz), 126.9 (2 C), 127.3 (2 C), 131.4, 145.6, 146.7 (d, *J* = 19.4 Hz), 148.0, 149.2, 151.4, 155.3, 158.9 (d, *J* = 12.8 Hz), 164.9 (d, *J* = 229.2 Hz) ppm. ^19^F NMR (DMSO-d6, 282 MHz) δ = -70.89 ppm. HR-MS *m/z* calculated for C_19_H_16_O_4_N_6_FS^-^ [M-H]^-^ 443.09433, found 443.09483, calculated for C_38_H_33_O_8_N_12_F_2_S_2_^-^ [2M-H]^-^ 887.19593, found 887.19637.

##### 8-(4-((4-Fluoropiperidin-1-yl)sulfonyl)phenyl)-1-propyl-3,7-dihydro-1*H*-purine-2,6-dione (**6**)

In 3 mL DMF, 150 mg (0.32 mmol, 1.0 eq.) **4**, 690 mg (3.18 mmol, 10.0 eq.) 4-fluoropiperidine TFA salt, and 466 µL (340 mg, 3.34 mmol, 10.5 eq.) triethylamine (TEA) were mixed and heated for 2.5 h at 125 °C. After the addition of 44 µL (32 mg, 0.32 mmol, 1.0 eq.) TEA and heating at 125 °C for 4 h, the reaction mixture was quenched with 250 mL 1M aq. HCl and extracted with 4 × 50 mL ethyl acetate. The combined organic layers were dried over sodium sulfate, filtered, and the solvent was removed under reduced pressure. The crude material was prepurified by column chromatography (ethyl acetate/*n*-hexane/acetic acid 1:2:0.1 *v*/*v*/*v*, silica gel, DCM/MeOH/HOAc 99:1:1 *v*/*v*/*v*, silica gel) to yield 61 mg (0.14 mmol, 44%) of **6**.

For binding studies, **6** was further purified via semipreparative HPLC (Reporsil-Pur Basic C18-HD, 5 µm, 20 × 250 mm, Dr. Maisch GmbH, Germany; 50% ACN/20 mM aq. ammonium acetate, flow rate of 6 mL/min; Jasco 2010Plus, LG-2080-04S, DG-2080-54, AS-2055Plus, LC-NetII/ADC; chromatograms were analyzed with the Galaxie Chromato-graphy Software (Agilent Technologies)).

R_f_ = 0.66 (DCM/MeOH/HOAc 99:1:1 *v*/*v*/*v*, silica gel). ^1^H NMR (DMSO-d_6_, 300 MHz) δ = 0.86 (t, *J* = 7.4 Hz, 3 H), 1.52–1.59 (m, 2 H), 1.76–1.93 (m, 4 H), 2.91–3.12 (m, 4 H), 3.80 (t, *J* = 7.2 Hz, 2 H), 4.72 (d, *J* = 47.3 Hz, 1 H), 7.85 (d, *J* = 8.5 Hz, 2 H), 8.31 (d, *J* = 8.5 Hz, 2 H), 11.99 (bs, 1 H) ppm. ^13^C NMR (DMSO-d6, 75 MHz) δ = 11.2, 21.0, 30.1 (d, *J* = 19.9 Hz, 2 C), 41.5, 42.2 (d, *J* = 6.2 Hz, 2 C), 86.7 (d, *J* = 169.3 Hz), 108.7, 127.0 (2 C), 128.1 (2 C), 133.1, 136.1, 147.5, 148.0, 151.0, 155.0 ppm. ^19^F NMR (DMSO-d6, 282 MHz) δ = −181.83 ppm. HR-MS *m/z* calculated for C_19_H_23_O_4_N_5_FS^+^ [M+H]^+^ 436.14493, found 436.14542; HR-MS *m/z* calculated for C_19_H_22_O_4_N_5_FSNa^+^ [M+Na]^+^ 458.12633, found 458.12774.

### 3.3. In Vitro Binding Assays: Determination of K_i_ Values

Membrane preparations of recombinant CHO or HEK cells expressing the respective human adenosine receptor subtype were obtained according to Borrman et al. [13] or purchased from Perkin Elmer (Waltham, MA, USA). Radioligand binding assays for human A_1_, A_2A_, A_2B_, and A_3_ receptors were performed according to Alnouri et al. [39]. The following radioligands were employed: [^3^H]2-chloro-*N*^6^-cyclopentyladenosine ([^3^H]CCPA, 1 nM) [40] for the A_1_ receptor, [^3^H]3-(3-hydroxypropyl)-7-methyl-8-(*m*-methoxystyryl)-1-propargylxanthine ([^3^H]MSX-2, 1 nM) [41] for the A_2A_ receptor, [^3^H]8-(4-[4-(4-chlorophenyl)piperazine-1-sulfonyl]phenyl)-1-propyl-2,3,6,7-tetrahydro-1*H*-purine-2,6-dione ([^3^H]PSB-603, 0.3 nM) [13] for the A_2B_ receptor, and [^3^H](*R*)-8-ethyl-4-methyl-2-(phenyl)1,4,7,8-tetrahydro-5*H*-imidazo[2,1-*i*]purin-5-one ([^3^H]PSB-11, 1 nM) [42] for the A_3_ receptor. Three separate experiments were performed for determination of *K*_i_ values. All data were analyzed with GraphPad Prism, version 4.1 (GraphPad Inc., La Jolla, CA).

## 4. Conclusions

In conclusion, a biodistribution study with [^3^H]PSB-603 resulted in a low brain uptake with an SUV of 0.2. Therefore, the structure of the lead compound PSB-603 was modified to achieve better BBB penetration by reducing the molecular size and the lipophilicity. Furthermore, fluorine was introduced into the molecule to allow the possibility for future radiolabeling with fluorine-18. Both novel fluorinated compounds **5** and **6** showed high binding affinity towards the A_2B_ receptor (*K*_i_ values of about 10 nM) and moderate selectivity towards the other adenosine receptor subtypes (A_1_, A_2A_, and A_3_). These findings provide a good basis for further modifications of the ligand structure to enhance selectivity towards the adenosine receptor subtypes with respect to future brain PET imaging of the adenosine A_2B_ receptor.

## Figures and Tables

**Table 2:** Determined *K*_i_ values of the novel fluorinated compounds **5** and **6** and the published *K*_i_ values of PSB-603, **7**, and **8** towards the four adenosine receptor subtypes, along with the corresponding calculated selectivity ratios.

Compd.	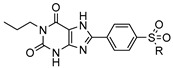	*K*_i_ in nM (or % Inhibition of Radioligand Binding at a Concentration of 1 µM) ^a^				Selectivity Ratio *K*_i_(A_x_)/*K*_i_(A_2B_)		
	R	A_2B_	A_2A_	A_1_	A_3_	A_2A_/A_2B_	A_1_/A_2B_	A_3_/A_2B_
**5**	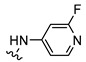	9.97 ± 0.86	375 ± 53	255 ± 32	~1000 (45 ± 3%)	38	25	100
**6**	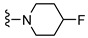	12.3 ± 3.6	158 ± 28	~1000 (44 ± 1%)	>1000 (38 ± 2%)	13	81	>81
PSB-603	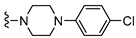	0.55 ± 0.10 ^b^	>10,000 (7 ± 14%) ^b^	>10,000 (10 ± 3%) ^b^	>10,000 (10 ± 15%) ^b^	>18,000	>18,000	>18,000
**7**	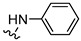	31.4 ± 0.9 ^b,c^	23 ± 13 ^b,d^	1.8 ± 1.0 ^b,d^	>1000 (48%) ^b^	0.7	0.06	>32
**8**	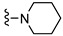	19.7 ± 4.7 ^b^	714 ± 122 ^b,d^	91.1 ± 25.0 ^b,d^	140 ± 20 ^b^	35	4	7

^a^ Data represent mean values ± SEM of three independent experiments that were performed on human receptors unless otherwise noted. Competition binding assays with [^3^H]PSB-603 (A_2B_), [^3^H]MSX-2 (A_2A_), [^3^H]CCPA (A_1_), and [^3^H]PSB-11 (A_3_) as radioligands and membranes were obtained from CHO cells stably expressing the corresponding human adenosine receptor. ^b^ Data are taken from [13,23,37]. ^c^ Determined with [^3^H]PSB-298. ^d^ Receptor binding determined with adenosine-receptor-expressing rat brain cortical membranes.

## Data Availability

The datasets generated and/or analyzed during the current study are available from the corresponding author on reasonable request.

## Supplementary Materials

Supplementary materials are available online at https://www.mdpi.com/article/10.3390/ph14050485/s1, Syntheses description for the precursor compound **4** and ^1^H, ^13^C, and ^19^F NMR and MS spectra for compounds **5** and **6** (Figures S1–S8). Figure S1: ^1^H NMR spectrum of compound **5**, Figure S2: ^13^C NMR spectrum of compound **5**, Figure S3: ^19^F NMR spectrum of compound **5**, Figure S4: Mass spectrum of compound **5**, Figure S5: ^1^H NMR spectrum of compound **6**, Figure S6: ^13^C NMR spectrum of compound **6**, Figure S7: ^19^F NMR spectrum of compound **6**, Figure S8: Mass spectrum of compound **6** and Scheme S1: Synthesis of xanthine backbone **4**.

## References

[1] Feoktistov I., Biaggioni I. (1997). Adenosine A_2B_ receptors. Pharmacol. Rev..

[2] Fredholm B.B., Abbracchio M.P., Burnstock G., Daly J.W., Harden T.K., Jacobson K.A., Leff P., Williams M. (1994). Nomenclature and classification of purinoceptors. Pharmacol. Rev..

[3] Fredholm B.B., Jzerman A.P., Jacobson K.A., Klotz K.-N., Linden J. (2001). International Union of Pharmacology. XXV. Nomenclature and classification of adenosine receptors. Pharmacol. Rev..

[4] Fredholm B.B., Jzerman A.P., Jacobson K.A., Linden J., Müller C.E. (2011). International Union of Basic and Clinical Pharmacology. LXXXI. Nomenclature and classification of adenosine receptors—An update. Pharmacol. Rev..

[5] Fredholm B.B., Irenius E., Kull B., Schulte G. (2001). Comparison of the potency of adenosine as an agonist at human adenosine receptors expressed in Chinese hamster ovary cells. Biochem. Pharmacol..

[6] Balakumar C., Sara S., Abdul Muttaleb Yousef J., Ghadir K., Nikhil A. (2019). Therapeutic Potentials of A_2B_ Adenosine Receptor Ligands: Current Status and Perspectives. Curr. Pharm. Des..

[7] Gao Z.-G., Jacobson K.A. (2019). A_2B_ Adenosine Receptor and Cancer. Int. J. Mol. Sci..

[8] Müller C.E., Baqi Y., Hinz S., Namasivayam V., Borea P.A., Varani K., Gessi S., Merighi S., Vincenzi F. (2018). Medicinal Chemistry of A_2B_ Adenosine Receptors. The Adenosine Receptors.

[9] Vecchio E.A., White P.J., May L.T. (2019). The adenosine A_2B_ G protein-coupled receptor: Recent advances and therapeutic implications. Pharmacol. Ther..

[10] Sarapynbiang M., Bijayashree M., Utpal C.D., Pratap C.A. (2019). Recent Progress of Adenosine Receptor Modulators in the Development of Anticancer Chemotherapeutic Agents. Curr. Pharm. Des..

[11] Kazemi M.H., Raoofi Mohseni S., Hojjat-Farsangi M., Anvari E., Ghalamfarsa G., Mohammadi H., Jadidi-Niaragh F. (2018). Adenosine and adenosine receptors in the immunopathogenesis and treatment of cancer. J. Cell. Physiol..

[12] Müller C.E., Jacobson K.A. (2011). Recent developments in adenosine receptor ligands and their potential as novel drugs. Biochim. Biophys. Acta Biomembr..

[13] Borrmann T., Hinz S., Bertarelli D.C.G., Li W., Florin N.C., Scheiff A.B., Müller C.E. (2009). 1-Alkyl-8-(piperazine-1-sulfonyl)phenylxanthines: Development and characterization of adenosine A_2B_ receptor antagonists and a new radioligand with subnanomolar affinity and subtype specificity. J. Med. Chem..

[14] Tanaka Y., Kitabatake K., Abe R., Tsukimoto M. (2020). Involvement of A_2B_ Receptor in DNA Damage Response and Radiosensitizing Effect of A_2B_ Receptor Antagonists on Mouse B16 Melanoma. Biol. Pharm. Bull..

[15] Kitabatake K., Yoshida E., Kaji T., Tsukimoto M. (2020). Involvement of adenosine A_2B_ receptor in radiation-induced translocation of epidermal growth factor receptor and DNA damage response leading to radioresistance in human lung cancer cells. Biochim. Biophys. Acta Gen. Subj..

[16] Petroni D., Giacomelli C., Taliani S., Barresi E., Robello M., Daniele S., Bartoli A., Burchielli S., Pardini S., Salvadori P.A. (2016). Toward PET imaging of A_2B_ adenosine receptors: A carbon-11 labeled triazinobenzimidazole tracer: Synthesis and imaging of a new A_2B_ PET tracer. Nucl. Med. Biol..

[17] Lindemann M., Hinz S., Deuther-Conrad W., Namasivayam V., Dukic-Stefanovic S., Teodoro R., Toussaint M., Kranz M., Juhl C., Steinbach J. (2018). Radiosynthesis and in vivo evaluation of a fluorine-18 labeled pyrazine based radioligand for PET imaging of the adenosine A_2B_ receptor. Bioorg. Med. Chem..

[18] Lindemann M., Moldovan R.-P., Hinz S., Deuther-Conrad W., Gündel D., Dukic-Stefanovic S., Toussaint M., Teodoro R., Juhl C., Steinbach J. (2020). Development of a Radiofluorinated Adenosine A_2B_ Receptor Antagonist as Potential Ligand for PET Imaging. Int. J. Mol. Sci..

[19] Kim Y.-C., Ji X.-d., Melman N., Linden J., Jacobson K.A. (2000). Anilide derivatives of an 8-phenylxanthine carboxylic congener are highly potent and selective antagonists at human A_2B_ adenosine receptors. J. Med. Chem..

[20] Kalla R.V., Elzein E., Perry T., Li X., Palle V., Varkhedkar V., Gimbel A., Maa T., Zeng D., Zablocki J. (2006). Novel 1,3-disubstituted 8-(1-benzyl-1*H*-pyrazol-4-yl) xanthines:  High affinity and selective A_2B_ adenosine receptor antagonists. J. Med. Chem..

[21] Baraldi P.G., Tabrizi M.A., Preti D., Bovero A., Romagnoli R., Fruttarolo F., Zaid N.A., Moorman A.R., Varani K., Gessi S. (2004). Design, synthesis, and biological evaluation of new 8-heterocyclic xanthine derivatives as highly potent and selective human A_2B_ adenosine receptor antagonists. J. Med. Chem..

[22] Ortore G., Martinelli A. (2010). A_2B_ receptor ligands: Past, present and future trends. Curr. Top. Med. Chem..

[23] Jiang J., Seel C.J., Temirak A., Namasivayam V., Arridu A., Schabikowski J., Baqi Y., Hinz S., Hockemeyer J., Müller C.E. (2019). A_2B_ Adenosine Receptor Antagonists with Picomolar Potency. J. Med. Chem..

[24] Brust P., van den Hoff J., Steinbach J. (2014). Development of ^18^F-labeled radiotracers for neuroreceptor imaging with positron emission tomography. Neurosci. Bull..

[25] Pike W.V. (2016). Considerations in the development of reversibly binding PET radioligands for brain imaging. Curr. Med. Chem..

[26] Wenzel B., Liu J., Dukic-Stefanovic S., Deuther-Conrad W., Teodoro R., Ludwig F.-A., Chezal J.-M., Moreau E., Brust P., Maisonial-Besset A. (2019). Targeting cyclic nucleotide phosphodiesterase 5 (PDE5) in brain: Toward the development of a PET radioligand labeled with fluorine-18. Bioorganic Chem..

[27] van de Waterbeemd H., Camenisch G., Folkers G., Chretien J.R., Raevsky O.A. (1998). Estimation of blood-brain barrier crossing of drugs using molecular size and shape, and H-bonding descriptors. J. Drug Target.

[28] Rankovic Z. (2015). CNS drug design: Balancing physicochemical properties for optimal brain exposure. J. Med. Chem..

[29] Waterhouse R.N. (2003). Determination of lipophilicity and its use as a predictor of blood-brain barrier penetration of molecular imaging agents. Mol. Imaging Biol..

[30] Rees D., Scanlon M., Ham J. (2003). Adenosine signalling pathways in the pituitary gland: One ligand, multiple receptors. J. Endochrinol..

[31] Dixon A.K., Gubitz A.K., Sirinathsinghji D.J.S., Richardson P.J., Freeman T.C. (1996). Tissue distribution of adenosine receptor mRNAs in the rat. Br. J. Pharmacol..

[32] Müller C.E. (1991). Synthesis of 3-substituted 6-aminouracils. Tetrahedron Lett..

[33] Müller C.E., Sandoval-Ramírez J. (1995). A new versatile synthesis of xanthines with variable substituents in the 1-, 3-, 7- and 8-positions. Synthesis.

[34] Müller C.E. (1993). General synthesis and properties of 1-monosubstituted xanthines. Synthesis.

[35] Yan L., Müller C.E. (2004). Preparation, properties, reactions and adenosine receptor affinities of sulfophenylxanthine nitrophenyl esters:  toward the development of sulfonic acid prodrugs with peroral bioavailability. J. Med. Chem..

[36] Lindemann M., Deuther-Conrad W., Moldovan R., Sekhar K.V.G.C., Brust P., Wenzel B. (2017). Do spiroindolines have the potential to replace vesamicol as lead compound for the development of radioligands targeting the vesicular acetylcholine transporter?. Bioorganic Med. Chem..

[37] Yan L., Bertarelli D.C.G., Hayallah A.M., Meyer H., Klotz K.-N., Müller C.E. (2006). A new synthesis of sulfonamides by aminolysis of *p*-nitrophenylsulfonates yielding potent and selective adenosine A_2B_ receptor antagonists. J. Med. Chem..

[38] Kanaya N., Ishiyama T., Muto R., Watanabe T., Ochiai Y. (2007). Pyrazole Derivatives.

[39] Alnouri M., Jepards S., Casari A., Schiedel A., Hinz S., Müller C. (2015). Selectivity is species-dependent: Characterization of standard agonists and antagonists at human, rat, and mouse adenosine receptors. Purinergic Signal..

[40] Klotz K.-N., Lohse M.J., Schwabe U., Cristalli G., Vittori S., Grifantini M. (1989). 2-Chloro-N^6^-[^3^H]cyclopentyladenosine ([^3^HCCPA) —A high affinity agonist radioligand for A_1_ adenosine receptors. Naunyn-Schmiedeberg’s Arch. Pharmacol..

[41] Müller C.E., Maurinsh J., Sauer R. (2000). Binding of [^3^H]MSX-2 (3-(3-hydroxypropyl)-7-methyl-8-(*m*-methoxystyryl)-1-propargylxanthine) to rat striatal membranes—A new, selective antagonist radioligand for A_2A_ adenosine receptors. Eur. J. Pharm. Sci..

[42] Müller C.E., Diekmann M., Thorand M., Ozola V. (2002). [^3^H]8-Ethyl-4-methyl-2-phenyl-(8*R*)-4,5,7,8-tetrahydro-1*H*-imidazo[2,1-*i*]-purin-5-one ([^3^H]PSB-11), a Novel High-Affinity Antagonist Radioligand for Human A_3_ Adenosine Receptors. Bioorg. Med. Chem. Lett..

