# Supplementary material for: Synthesis of Novel Fluorinated Xanthine Derivatives with High Adenosine A2B Receptor Binding Affinity
Source: Pharmaceuticals (Basel). 2021 May 19;14(5):485. doi: 10.3390/ph14050485 (PMC8161391; doi:10.3390/ph14050485)
Supplement: Supplementary file 1 [file pharmaceuticals-14-00485-s001.zip › pharmaceuticals-1210115-supplementary.pdf]

Supporting Information to

Synthesis of novel fluorinated xanthine derivatives with high adenosine A_{2B} receptor binding affinity

Marcel Lindemann[1*], Sladjana Dukic-Stefanovic[1,2], Sonja Hinz[3], Winnie Deuther-Conrad[1], Rodrigo Teodoro[1], Cathleen Juhl[2], Jörg Steinbach[1], Peter Brust[1], Christa E. Müller[3], Barbara Wenzel[1]

1 Helmholtz-Zentrum Dresden-Rossendorf, Institute of Radiopharmaceutical Cancer Research, Department of Neuroradiopharmaceuticals, Leipzig, Germany; marcel.lindemann.cor@gmail.com (M.L.), s.dukic-stefanovic@hzdr.de (S.D.-S.), w.deuther-conrad@hzdr.de (W.D.-C.), r.teodoro@hzdr.de (R.T.), Steinbach-joerg@web.de (J.S.), p.brust@hzdr.de (P.B.), b.wenzel@hzdr.de (B.W.)

2 ROTOP Pharmaka GmbH, Dresden, Germany; service@rotop-pharmaka.de (S.D.S. and C.J.)

3 Pharma Center Bonn, Pharmaceutical Institute, Pharmaceutical & Medicinal Chemistry, University of Bonn, Bonn, Germany; Sonja.Hinz@uni-wh.de (S.H.), christa.mueller@uni-bonn.de (C.E.M.)

* Correspondence: marcel.lindemann.cor@gmail.com

Content:

1. Synthesis of the precursor compound **4** 2
2. ^{1}H, ^{13}C, ^{19}F NMR spectra and mass spectra of compounds **5** and **6** .... 3 - 6
3. References 7

1. Synthesis of the precursor compound 4

Starting with 6-aminouracil **9** in a selective *N*-alkylation [1,2], nitrosylation and reduction with hydrogen and palladium on carbon to the diamine **11** was performed [2,3]. The 4-((4-nitrophenoxy)sulfonyl)benzoic acid was synthesized over a two-step approach starting from potassium 4-sulfobenzoate, which was chlorinated and converted to the final sulfonyl ester [4]. Instead of using 1-ethyl-3-(3-dimethylaminopropyl)carbodiimide (EDC) [4], benzotriazolooxytris(dimethylamino)phosphoniumhexafluorophosphat (BOP) was used for the formation of the amide **12**, because it already showed good results in another study [5]. The reaction was performed in dichloromethane (DCM) with triethylamine (TEA) as base, to result in **12** with moderate yields of around 36%. The obtained amide **12** was intramolecular cyclized to result in the xanthine backbone precursor **4** with a yield of 91% [4].

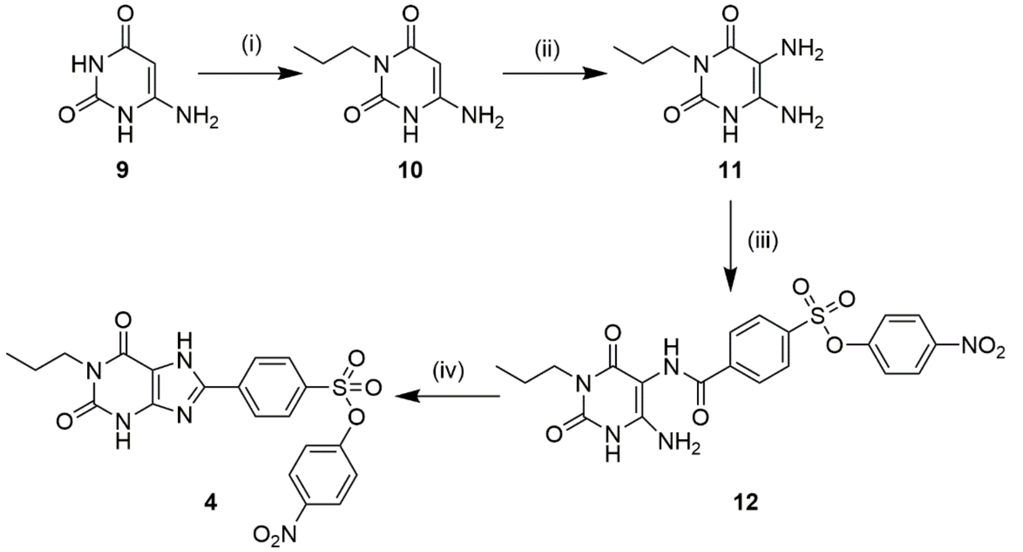

Scheme S1. Synthesis of xanthine backbone **4**. Reaction conditions: (i) a) cat. $(NH_4)_2SO_4$, 1,1,1,3,3,3-hexamethyldisilazan, reflux, 2 h, b) toluene, 1-iodopropane, reflux, 16 h, c) sat. aq. $NaHCO_3$, 71%; (ii) a) 50% aq. HOAc, $NaNO_2$, 70 °C, 30 min, b) H_2/Pd/C, ethanol, room temperature, 5 h, 85%; (iii) BOP, TEA, 4-((4-nitrophenoxy)sulfonyl)benzoic acid, DCM, room temperature, 2 d, 36%; (iv) polyphosphoric acid trimethylsilyl ester, 145 °C, 8 h, 91%.

2. ^{1}H, ^{13}C, ^{19}F NMR and mass spectra of compounds 5 and 6

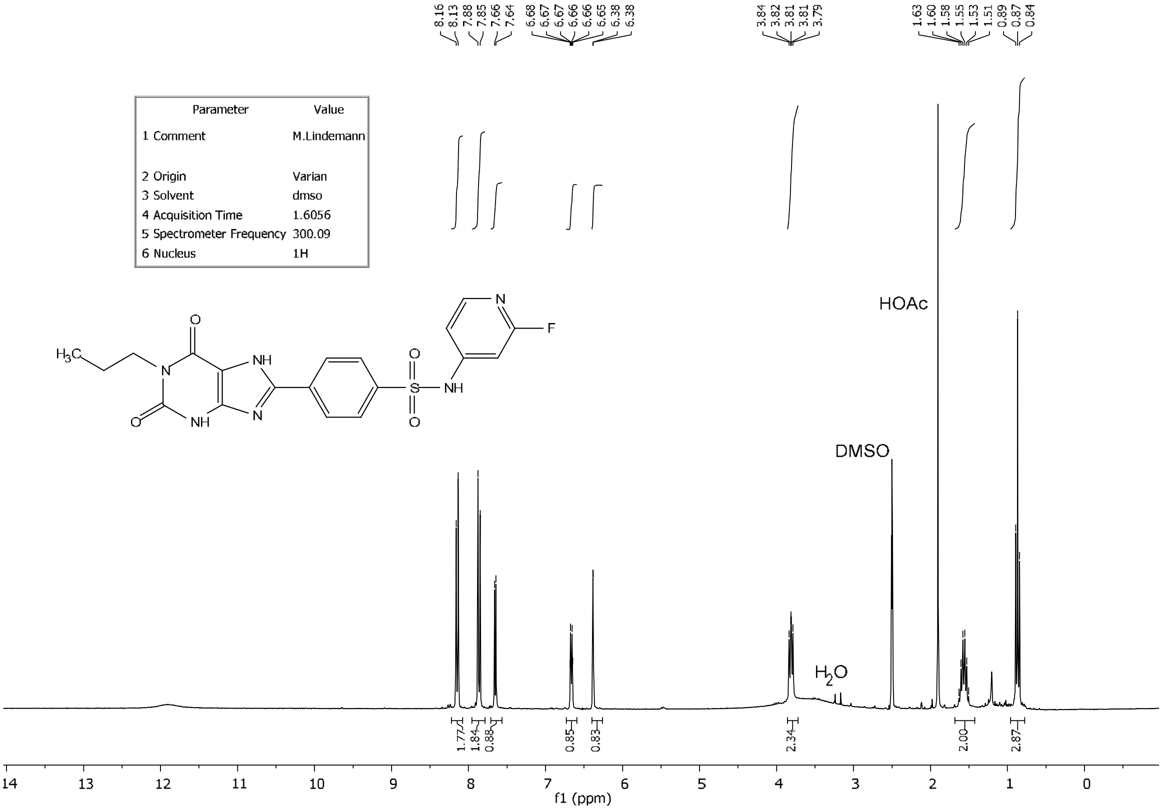

Figure S1. ^{1}H NMR spectrum of compound **5**.

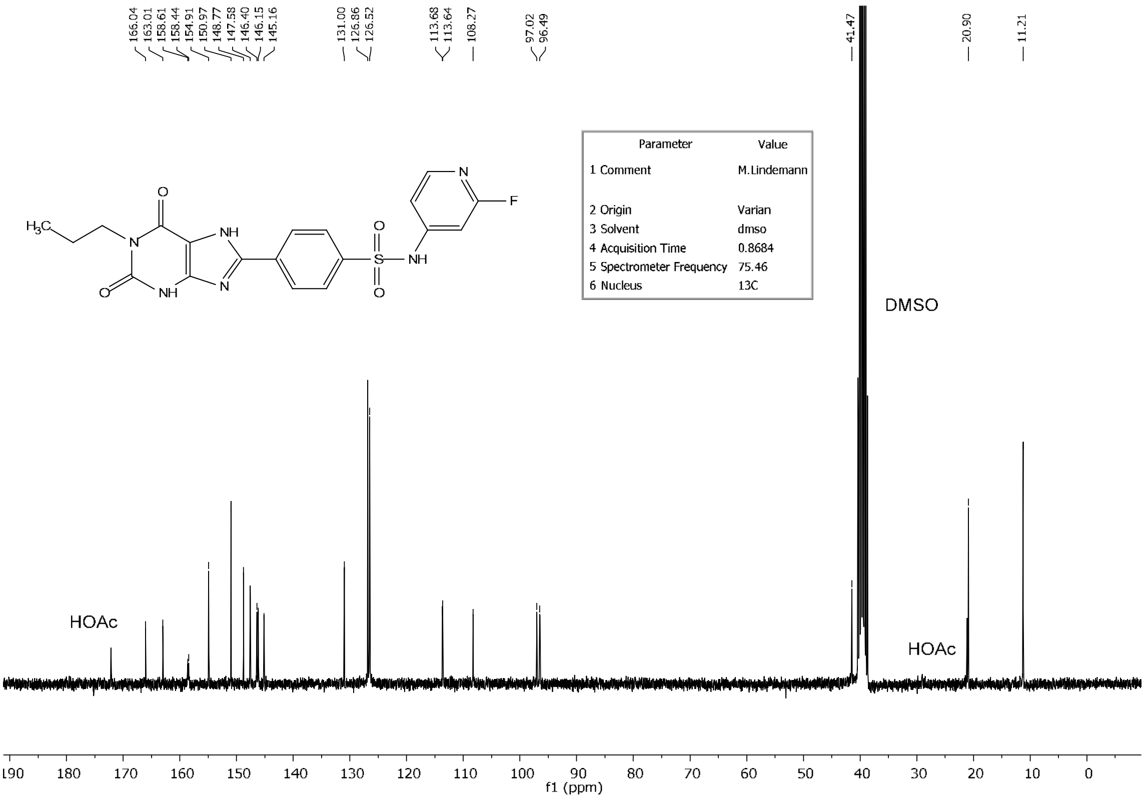

Figure S2. ^{13}C NMR spectrum of compound **5**.

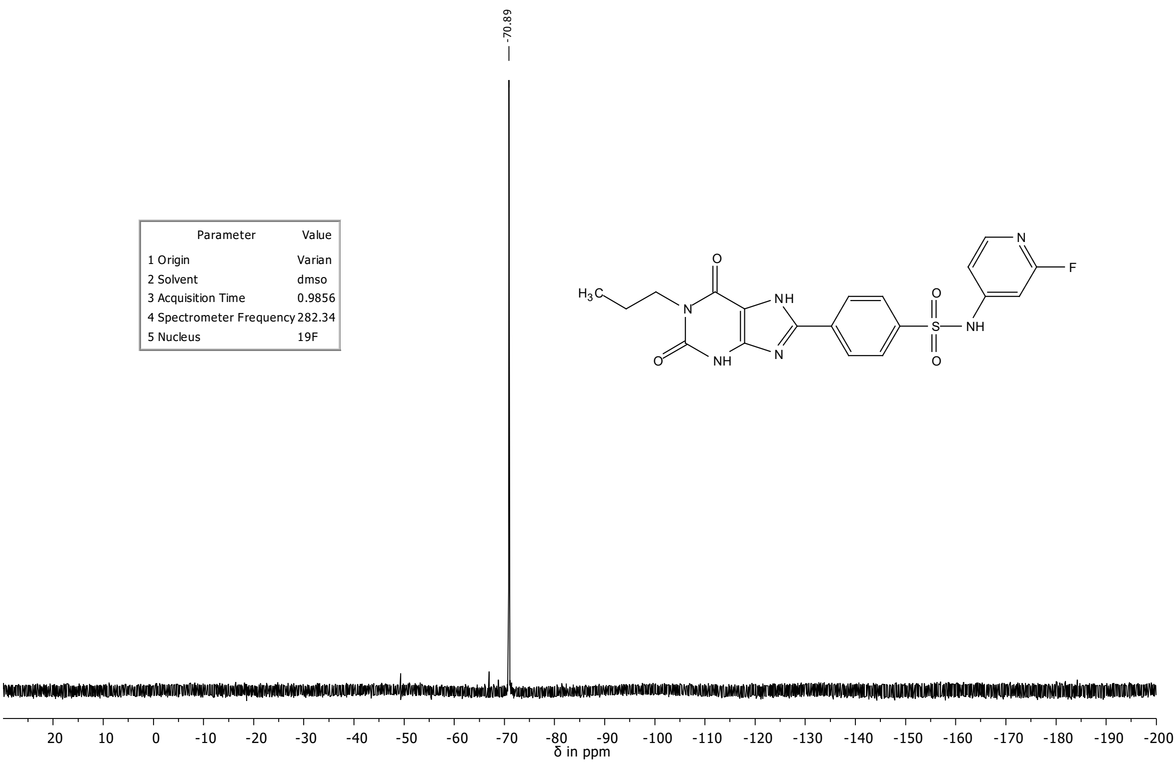

Figure S3. ^{19}F NMR spectrum of compound **5**.

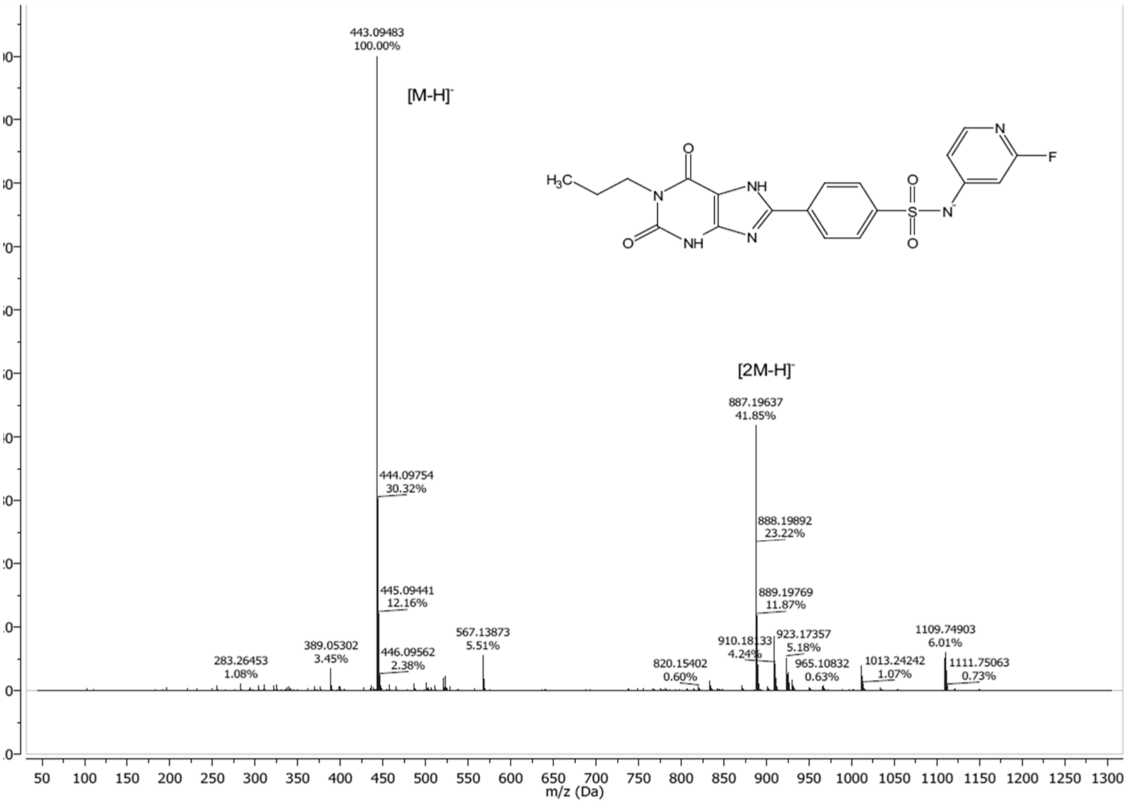

Figure S4. Mass spectrum of compound **5**.

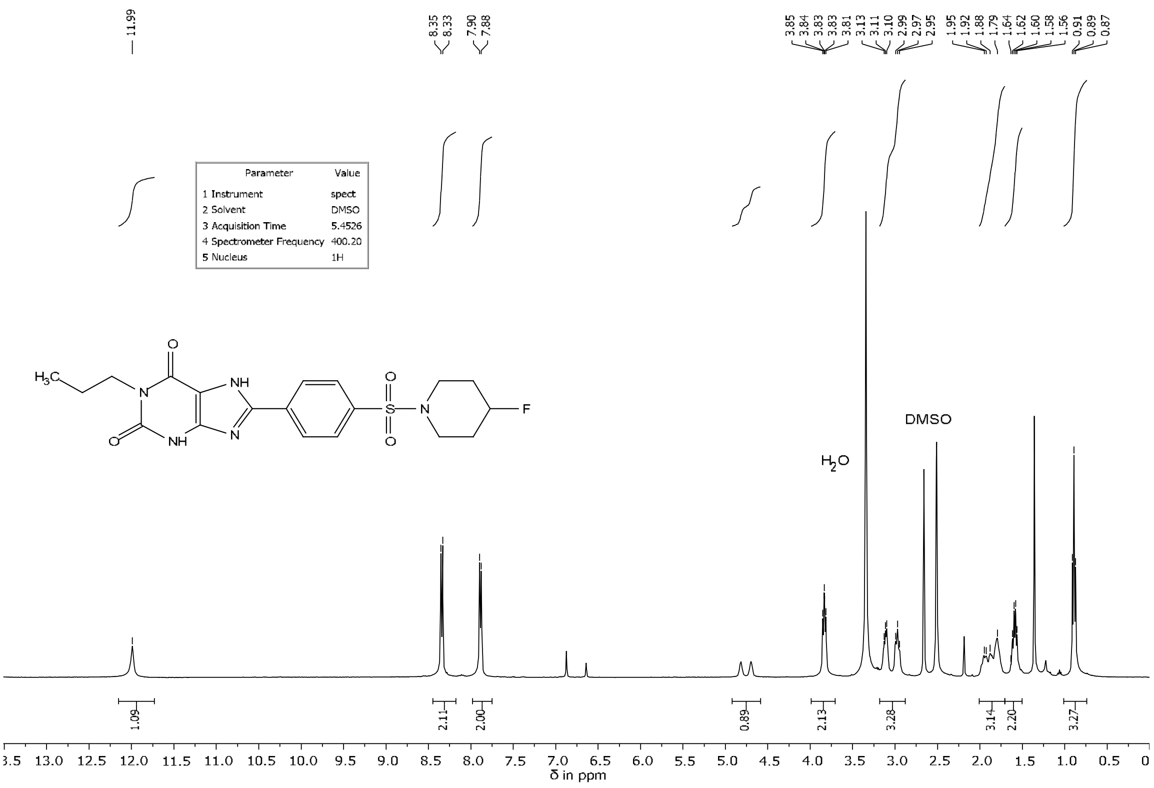

Figure S5. ^{1}H NMR spectrum of compound **6**.

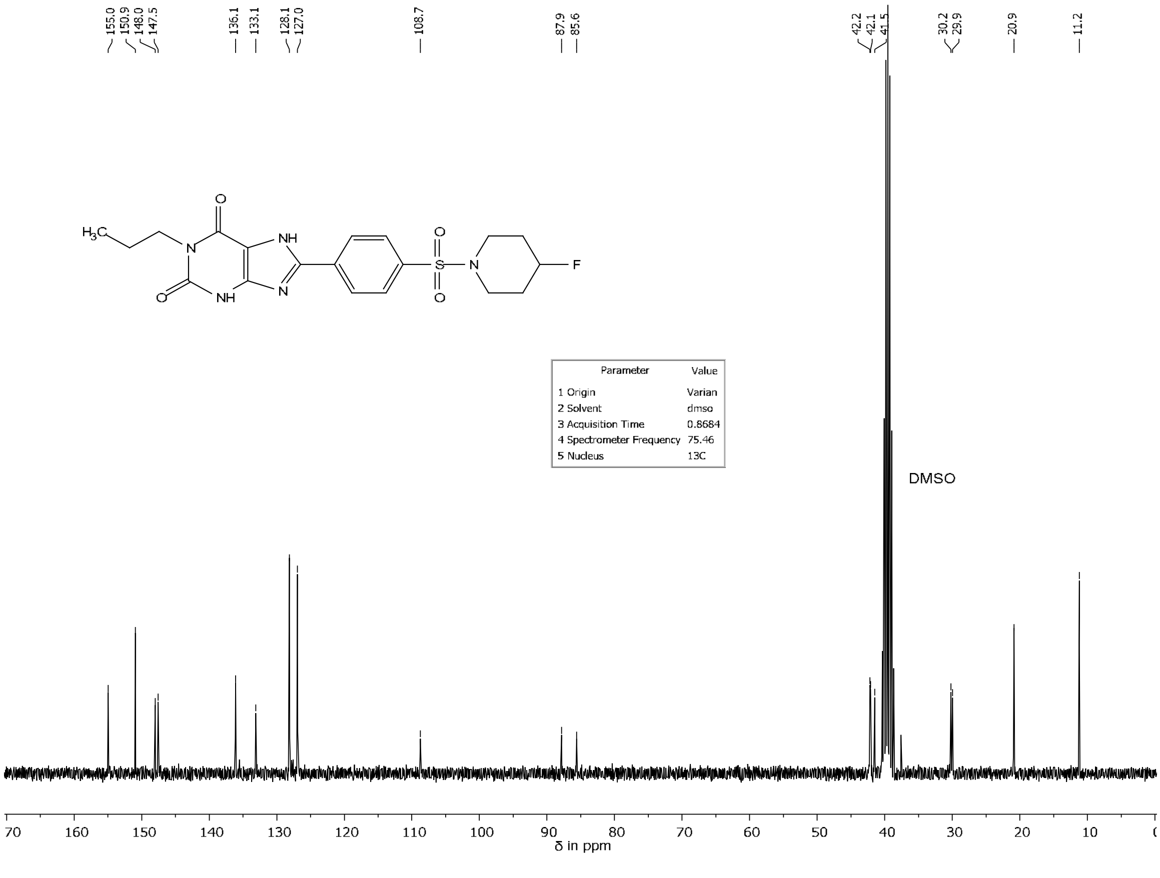

Figure S6. ^{13}C NMR spectrum of compound **6**.

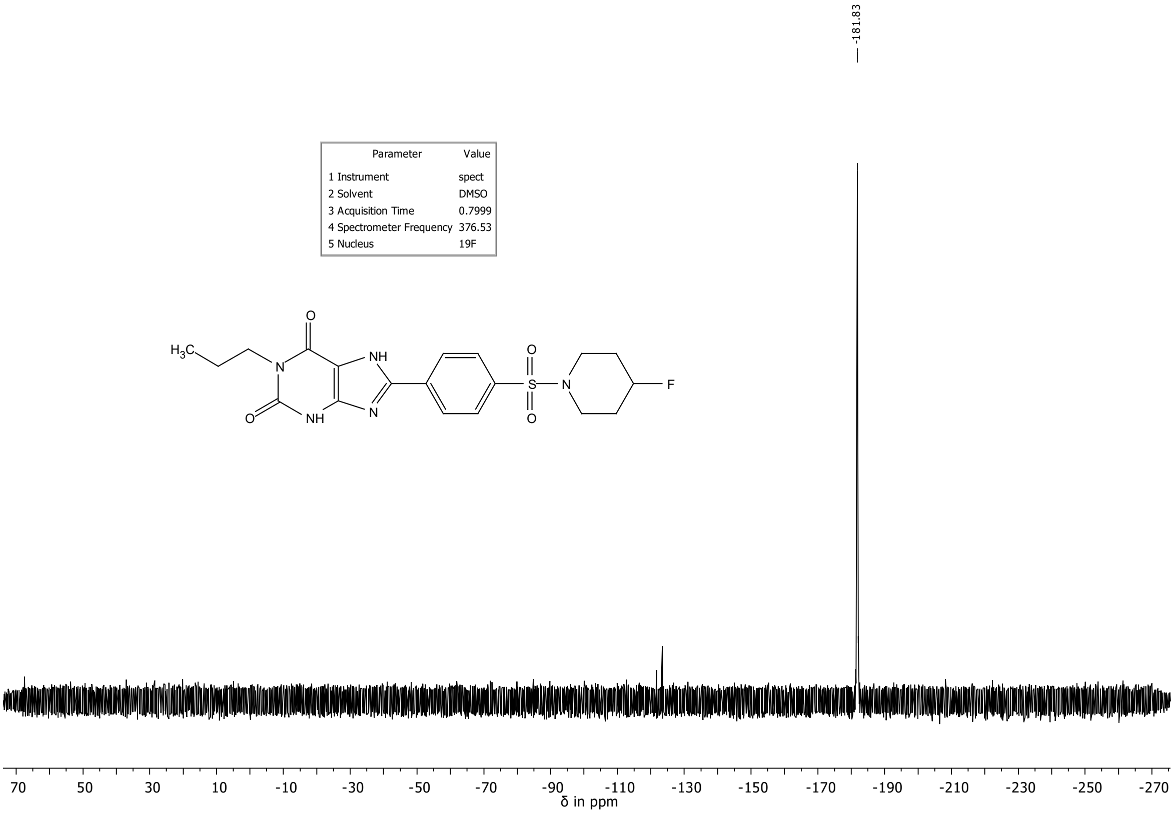

Figure S7. ^{19}F NMR spectrum of compound **6**.

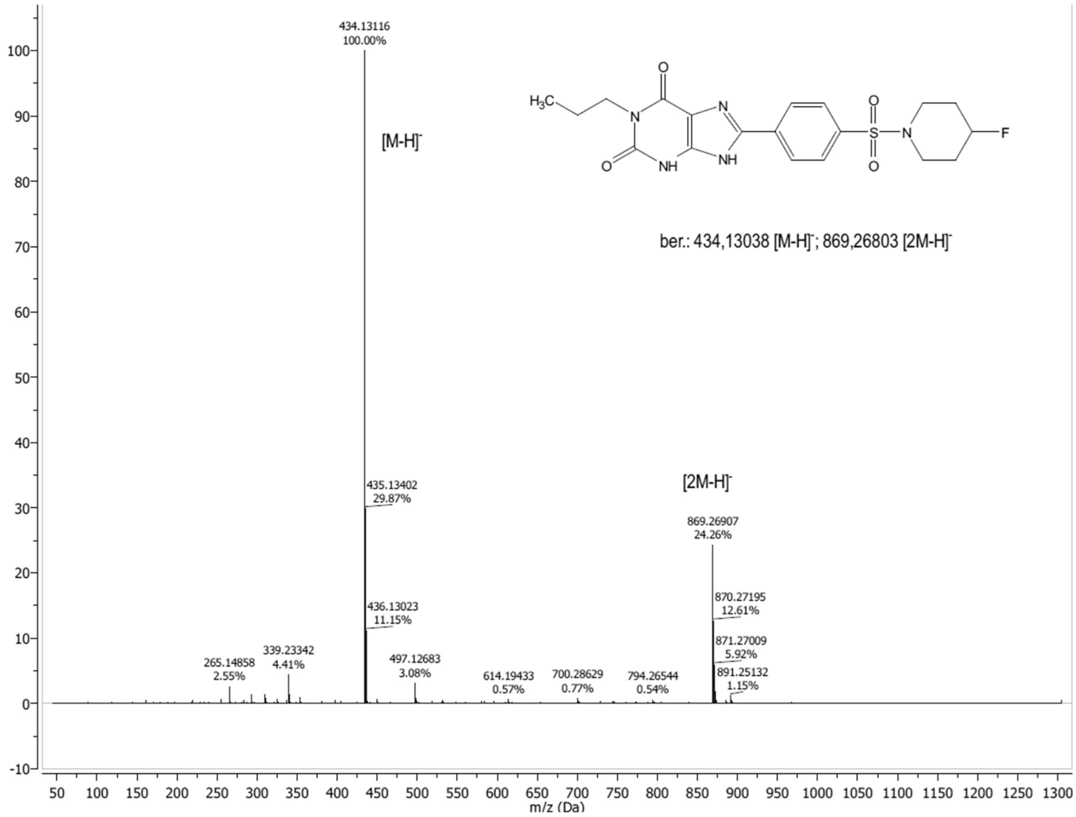

Figure S8. Mass spectrum of compound **6**.

3. References

1. Müller, C.E. Synthesis of 3-substituted 6-aminouracils. *Tetrahedron Lett.* **1991**, *32*, 6539-6540, doi:http://dx.doi.org/10.1016/0040-4039(91)80214-Q.
2. Müller, C.E.; Sandoval-Ramírez, J. A new versatile synthesis of xanthines with variable substituents in the 1-, 3-, 7- and 8-positions. *Synthesis* **1995**, *1995*, 1295-1299, doi:10.1055/s-1995-4082.
3. Müller, C.E. General synthesis and properties of 1-monosubstituted xanthines. *Synthesis* **1993**, *1993*, 125-128, doi:10.1055/s-1993-25814.
4. Yan, L.; Müller, C.E. Preparation, properties, reactions and adenosine receptor affinities of sulfophenylxanthine nitrophenyl esters: toward the development of sulfonic acid prodrugs with peroral bioavailability. *J. Med. Chem.* **2004**, *47*, 1031-1043, doi:10.1021/jm0310030.
5. Lindemann, M.; Deuther-Conrad, W.; Moldovan, R.; Sekhar, K.V.G.C.; Brust, P.; Wenzel, B. Do spiroindolines have the potential to replace vesamicol as lead compound for the development of radioligands targeting the vesicular acetylcholine transporter? *Bioorg. Med. Chem.* **2017**, *25*, 5107-5113, doi:https://doi.org/10.1016/j.bmc.2017.03.028.
